# Critical region within 22q11.2 linked to higher rate of autism spectrum disorder

**DOI:** 10.1186/s13229-017-0171-7

**Published:** 2017-10-27

**Authors:** Caitlin C. Clements, Tara L. Wenger, Alisa R. Zoltowski, Jennifer R. Bertollo, Judith S. Miller, Ashley B. de Marchena, Lauren M. Mitteer, John C. Carey, Benjamin E. Yerys, Elaine H. Zackai, Beverly S. Emanuel, Donna M. McDonald-McGinn, Robert T. Schultz

**Affiliations:** 1 Center for Autism Research, The Children’s Hospital of Philadelphia, 2716 South Street, Philadelphia, PA 19104 USA; 2 Department of Psychology, University of Pennsylvania, 3720 Walnut Street, Philadelphia, PA 19104 USA; 3 Department of Pediatrics, Seattle Children’s Hospital, 4800 Sand Point Way NE, Seattle, WA 98105 USA; 4 Vanderbilt Brain Institute, Vanderbilt University School of Medicine, 465 21st Avenue South, Nashville, TN 37232 USA; 5 Department of Psychiatry, University of Pennsylvania, 3400 Civic Center Boulevard, Philadelphia, PA 19104 USA; 6 Department of Behavioral and Social Science, University of the Sciences, 600 South 43rd Street, Philadelphia, PA 19104 USA; 7 Department of Pediatrics, The Children’s Hospital of Philadelphia, 3400 Civic Center Boulevard, Philadelphia, PA 19104 USA; 8 Department of Pediatrics, University of Utah, Salt Lake City, UT 84108 USA

**Keywords:** 22q11.2 deletion syndrome, 22q11.2 duplication syndrome, Autism spectrum disorder, *RANBP1*, Screening, Atypical, Nested, Syndromic autism, Prosopagnosia, Face processing

## Abstract

**Background:**

Previous studies have reported no clear critical region for medical comorbidities in children with deletions or duplications of 22q11.2. The purpose of this study was to evaluate whether individuals with small nested deletions or duplications of the LCR-A to B region of 22q11.2 show an elevated rate of autism spectrum disorder (ASD) compared to individuals with deletions or duplications that do not include this region.

**Methods:**

We recruited 46 patients with nested deletions (*n* = 33) or duplications (*n* = 13) of 22q11.2, including LCR-A to B (*n*_del_ = 11), LCR-A to C (*n*_del_ = 4), LCR-B to D (*n*_del_ = 14; *n*_dup_ = 8), LCR-C to D (*n*_del_ = 4; *n*_dup_ = 2), and smaller nested regions (*n* = 3). Parent questionnaire, record review, and, for a subset, in-person evaluation were used for ASD diagnostic classification. Rates of ASD in individuals with involvement of LCR-B to LCR-D were compared with Fisher’s exact test to LCR-A to LCR-B for deletions, and to a previously published sample of LCR-A to LCR-D for duplications. The rates of medical comorbidities and psychiatric diagnoses were determined from questionnaires and chart review. We also report group mean differences on psychiatric questionnaires.

**Results:**

Individuals with deletions involving LCR-A to B showed a 39–44% rate of ASD compared to 0% in individuals whose deletions did not involve LCR-A to B. We observed similar rates of medical comorbidities in individuals with involvement of LCR-A to B and LCR-B to D for both duplications and deletions, consistent with prior studies.

**Conclusions:**

Children with nested deletions of 22q11.2 may be at greater risk for autism spectrum disorder if the region includes LCR-A to LCR-B. Replication is needed.

**Electronic supplementary material:**

The online version of this article (10.1186/s13229-017-0171-7) contains supplementary material, which is available to authorized users.

## Background

22q11.2 deletion syndrome (22q11.2DS), also known as DiGeorge or velocardiofacial syndrome, is the most common microdeletion syndrome in humans. The 3 Mb region most frequently affected in 22q11.2DS can also be duplicated, resulting in 22q11.2 duplication syndrome (22q11.2DupS) [1]. Previous studies suggested a prevalence of 1 in 4000 live births for 22q11.2DS, but a recent study of consecutive pregnancies found an incidence of 1 in 992 live births––similar to that of trisomy 21 [2]. The incidence of 22q11.2DupS was found to be 1 in 850 [2].

**Fig. 1:**
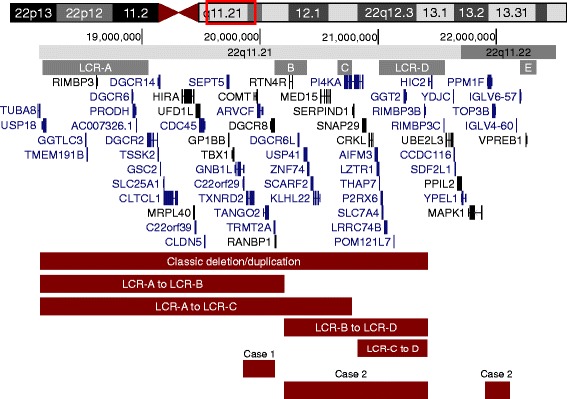
22q11.2 diagram. Genes and low-copy repeat (“LCR”) regions in the 22q11.2 region. Red bars depict deletions or duplications of participants. From GENCODE v24 genes in UCSC genome browser, December 2013 Assembly (genome.ucsc.edu)

The 22q11.2 region includes low copy repeats (LCRs or segmental duplication blocks) interspersed throughout the region that frequently result in “breakpoints” for a duplication or deletion. The most commonly duplicated or deleted region spans LCR-A to LCR-D (hereafter––“classic” deletion/duplication). However, smaller nested deletions occur in 15% of affected individuals and usually stretch from only LCR-A to LCR-B, or from LCR-B to LCR-D, but can also span LCR-A to LCR-C or LCR-C to LCR-D [3]. In other cases, deletions include the area upstream of LCR-A or extend past LCR-D (see Fig. 1). The diagnoses of 22q11.2DS or 22q11.2DupS can refer to patients with either a classic or nested deletion/duplication.

The phenotypes of 22q11.2DS and 22q11.2DupS overlap with one another and show significant individual differences [4, 5]. The syndromes can affect almost any organ system, and individuals can present with diverse constellations of medical issues and structural malformations, as well as a wide range of severity. Common medical comorbidities include congenital heart disease, hypocalcemia, renal abnormalities, immune deficiencies, and neuropsychiatric differences [1]. There is a recognizable facial gestalt in 22q11.2DS, but no recognizable gestalt has been identified in 22q11.2DupS. The rate of medical problems is much lower in 22q11.2DupS [6].

The 22q11.2 region has also been associated with elevated rates of autism spectrum disorder (from now on referred to as “ASD”), attention deficit/hyperactivity disorder (ADHD), and most notably, schizophrenia. A recent large study of 22q11.2DS reported psychosis in 41% of adults and ADHD in 37% of children [7], although a psychiatric registry-based study found lower rates [8]. Interestingly, there are no reported individuals with 22q11.2DupS with schizophrenia, and one group even suggested that it may be protective for schizophrenia [9]. In contrast, an elevated risk of ASD is found in both 22q11.2DS and 22q11.2DupS. As many as 50% of individuals with 22q11.2DS and 38% with 22q11.2DupS have received community diagnoses of autism spectrum disorder; however, fewer meet strict diagnostic criteria in research settings with reported rates of 0–18% in 22q11.2DS [10–12] and 14–25% in 22q11.2DupS [6].

Despite significant heterogeneity in the 22q11.2 phenotype [13], little is known about critical regions that may confer risk for any specific part of the phenotype beyond schizophrenia, cleft palate, and cardiac anomalies. Prior reports point to *TBX1*, *CRKL*, and *MAPK1* as contributors to the cardiac [14–18] and cleft palate phenotypes [19] in 22q11.2DS. Other research linked schizophrenia risk in 22q11.2DS to hyperprolinemia associated with lowered expression of proline dehydrogenase (*PRODH*) [20, 21]. Some studies reported an association between schizophrenia risk in 22q11.2DS and the lower activity Met allele of catechol-O-methyltransferase (*COMT*) [20, 22, 23], but larger cohort studies found no evidence [24–26] (for review, see [27]). These risk genes span the 22q11.2 region, with *COMT*, *PRODH*, and *TBX1* lying between LCR-A and LCR-B, while *CRKL* lies between LCR-C and LCR-D, and *MAPK1* lies between LCR-D and LCR-E.

Recent research identified two genes as potential mediators of the ASD risk in 22q11.2DS. Radoeva et al. reported that in a sample of 87 individuals with 22q11.2DS, individuals with ASD were more likely to carry both the low-activity alleles of *COMT* and *PRODH* (leading to high levels of proline) than individuals without ASD [28]. Neither gene individually showed a significant direct relationship with ASD, although the pattern trended in that direction. Hidding et al. further demonstrated a quantitative relationship between ASD symptom severity and the combination of *COMT*-Met genotype and high proline levels in 45 individuals with 22q11.2DS with and without ASD [29]. Both results suggest that the interaction between *COMT* and *PRODH*, which lie in the LCR-A to B region, may increase ASD risk in individuals with 22q11.2DS.

The purpose of the present study was to leverage a novel study design to determine whether risk for autism can be narrowed to the LCR-A to LCR-B region within 22q11.2. Owing to the rarity of these nested structural variants, this is the first study to our knowledge that attempts to collect and phenotype large enough samples to test this hypothesis. We hypothesized that individuals harboring deleted LCR-A to LCR-B would show higher rates of ASD [30]; in addition to this region harboring *COMT* and *PRODH*, it also contains *RANBPI*, a gene involved in the metabotropic glutamate receptor (mGluR) gene network that we previously hypothesized could play a role in ASD in 22q11.2DS/DupS [30]. In addition, we describe two case studies (one from our cohort and one from the literature) with much smaller, atypical duplications within the LCR-A to B region to gain hints as to the role of specific genes.

## Methods

### Participants

#### Participants with nested 22q11.2 duplications or deletions

Participants included 43 individuals with a nested duplication (*n* = 13) or deletion (*n* = 30) of 22q11.2 that lay entirely within LCR-A to LCR-D but was not completely inclusive of LCR-A to LCR-D (see Table 1). The only exception to this was one participant who carried a duplication of LCR-B to LCR-D and also a very small duplication between LCR-E and LCR-F. Participants were recruited from a specialty clinic at The Children’s Hospital of Philadelphia (CHOP) or were referred from a similar specialty clinic at another institution. The CHOP “22q and You” clinic represents the largest single-site 22q11.2 clinic in the world and maintains a large catchment area across the eastern US, with patients concentrated within a few hundred mile radius of CHOP. The sample includes probands who came to clinical attention, as well as their affected siblings (*n* = 2 with duplication and *n* = 3 with deletion) and parents (*n* = 2 with duplication and *n* = 2 with deletion) whose 22q11.2DS or 22q11.2DupS was identified after the proband’s diagnostic process. The duplication or deletion was confirmed using single nucleotide polymorphism (SNP) microarray or Multiplex Ligation Probe Amplification (MLPA).

**Table 1:** Descriptive characteristics of all participants in study

Region	Number	% de novo^a^	Age mean (sd)	Age range (years)	% male
Total	46	60%	10.8(10.1)	0.8–39	52%
AB/AC deletion group	15	86%	8.9(4.2)	2–15	53%
A-B deletion	11	80%	7.8(3.8)	2–15	55%
A-C deletion	4	100%	11.8(4.4)	5–14	50%
BD/CD deletion group	18	69%	11.1(10.7)	1–38	50%
B-D deletion	14	77%	10.4(9.0)	1–38	43%
C-D deletion	4	33%	13.6(16.9)	0.8–36	75%
BD/CD duplication group	10	13%	14.5(15.2)	1–39	60%
B-D duplication	8	0%	16.5(16.6)	1–39	63%
C-D duplication	2	50%	6.5(2.0)	5–7	50%
A-B duplication^b^	1	Unknown	7.0	–	0%
E-F duplication^b^	2	0%	6.6(2.8)	4–8	50%

Participant characteristics for all individuals with a nested deletion or duplication of 22q11.2, including three case studies with atypical nested duplications noted with superscript *b*

^a^Percentage of individuals with known inheritance

^b^Case studies not included in statistical analysis, medical chart review, or AB/AC and BD/CD group totals

Samples whose copy number variations (CNVs) were tested by MLPA were examined using the SALSA P250 DiGeorge diagnostic probe kit (MRC-Holland, Amsterdam, The Netherlands). Commercially available software, Gene Marker from SoftGenetics (State College, PA), was used to analyze the data. Gene Marker has developed a completely integrated application for MLPA analysis with integrated functions specific for the analysis of data derived from MLPA reactions. Samples whose CNVs were identified by SNP array were analyzed using the Affymetrix SNP Array 6.0 platform following the manufacturer’s instructions (Affymetrix, Santa Clara, CA, USA). Quality control values were calculated in Affymetrix Genotyping Console (Affymetrix), and any samples with contrast QC greater than 0.4 or mean absolute pairwise difference (MAPD) greater than 0.35 were excluded from further analysis. The B allele frequency and log R ratio plots were visualized using the Affymetrix Chromosome Analysis Suite to support CNV calls.

Three additional patients who carried very small and rare atypical duplications are included in this paper in a descriptive manner (in the case study sections), but are not combined with the other groups in tables, figures, or statistical analyses. One patient carried a very small duplication within LCR-A to LCR-B. The other two patients (who were related to three patients in the main LCR-B to D duplication group) carried a small duplication nested between LCR-E and LCR-F.

**Fig. 2:**
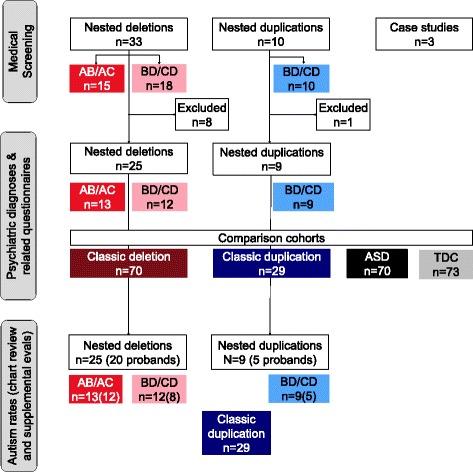
Participant flow chart depicting the participants and comparisons included in each portion of the study. Group colors correspond to colors in Figs. 3 and 4 and Additional file 2. Abbreviations: *AB/AC* deletion or duplication spanning LCR-A to LCR-B or LCR-A to LCR-C, *ASD* non-syndromic autism spectrum disorder, *BD/CD* deletion or duplication spanning LCR-B to LCR-D or LCR-C to LCR-D, *classic deletion* typical 22q11.2 deletion syndrome involving LCR-A to D, *classic duplication* typical 22q11.2 duplication syndrome involving LCR-A to D, *LCR* low-copy repeat region, *TDC* typically developing controls

All 43 participants were included in the medical history chart review. Nine participants were excluded from the ASD and psychiatric symptom analyses (*n* = 34; see Fig. 2) for two types of reasons: (1) ASD classification could not be determined (*n* = 2; see below) or (2) if they presented with another medical issue likely to affect brain development (*n* = 2 extreme prematurity and/or birth weight < 5th centile, *n* = 2 with CEDNIK syndrome, *n* = 1 with 16p11.2 deletion which is independently associated with ASD, *n* = 2 history of hypoxic brain injury) [31–34]. Participant characteristics of the sample excluding these nine cases are described in Table 2. Please note that some ages differ from those in the medical record review (Table 1) because a review of updated records pertinent to ASD classification, when available, was conducted 3 years later to allow for infants to reach the age (3 years) at which ASD symptoms would be present.

**Table 2:** Descriptive characteristics of participants included in psychiatric diagnosis rates

Region	Number	% de novo^a^	Age mean (sd)	Age range (years)	% male
AB/AC deletion group	13	83%	10 (4.2)	5–18	54%
A-B deletion	10	78%	8.9 (3.6)	5–15	60%
A-C deletion	3	100%	13.7 (4.8)	9–18	33%
BD/CD deletion group	12	50%	14.2 (12.7)	3–42	50%
B-D deletion	8	57%	13.4 (12.1)	4–42	38%
C-D deletion	4	33%	15.9 (15.5)	3–37	75%
BD/CD duplication group	9	14%	16.9 (14.7)	5–39	56%
B-D duplication	7	0%	19.4 (15.9)	5–39	57%
C-D duplication	2	50%	8 (4.1)	5–11	50%
Total	34	55%	13.3 (11.0)	3–42	53%

Participant characteristics for the subset of individuals with a nested deletion or duplication of 22q11.2 included in description of psychiatric diagnosis rates

^a^Percentage of individuals with known inheritance

Rates of autism were analyzed separately for individuals with nested deletions and duplications (see Table 3). Only one individual per family (the proband) was included to avoid confounding autism rates with risk factors shared by related individuals. In one family with B-D duplication, we included an affected family member instead of the proband because the proband harbored a 16p11.2 deletion. For deletions, 20 individuals were included after excluding five parents and younger siblings (2 B-D, 2 C-D, 1 A-B). For duplications, five individuals were included after excluding four parents and younger siblings (4 B-D). No individuals presented with nested duplications involving LCR-A to LCR-B or C.

**Table 3:** ASD rates among probands

Region	Number	*n* ASD (male)	% de novo^a^	Age mean (sd)	Age range (years)	% male
AB/AC deletion group	12	5(3)	90%	10.0 (4.4)	5–18	58%
A-B deletion	9	4(3)	90%	8.7 (3.7)	5–15	67%
A-C deletion	3	1(0)	100%	13.7 (4.8)	9–18	33%
BD/CD deletion group	8	0	60%	10.5 (4.8)	5–18	25%
B-D deletion	6	0	70%	10.2 (3.8)	6–17	17%
C-D deletion	2	0	0%	11.6 (9.1)	5–18	50%
Classic AD duplication	29	7(5)	67%	7.1 (3.4)	2–13	75%
BD/CD duplication group	5	1(0)	30%	12.7 (10.4)	5–31	60%
B-D duplication	3	1(0)	0%	15.8 (13.1)	7–31	67%
C-D duplication	2	0	50%	8.0 (4.1)	5–11	50%

Participant characteristics and autism diagnosis for all probands with a nested deletion of 22q11.2. Individuals harboring an AB or AC deletion presented with ASD at a rate of 41.6% (5 of 12). Case studies are excluded from this table

^a^Percentage of individuals with known inheritance

#### Comparison cohorts

We compiled comparison questionnaire data from four cohorts. Detailed results of medical system chart review, neuropsychiatric questionnaires, ASD symptoms, and adaptive functioning of these four comparison groups have been published elsewhere [6]. Two cohorts were drawn from patients at the same clinic who had a confirmed classic (LCR-A to LCR-D) 22q11.2 duplication (*n* = 29) or deletion (*n* = 70). A non-syndromic ASD cohort (*n* = 70) and typically developing control cohort (*n* = 73) were drawn from other studies of neurodevelopment at the CHOP Center for Autism Research. These four cohorts were age- and sex-matched to one another but were not as well matched to either of the small nested samples described above to allow for inclusion of all eligible individuals with a nested CNV.

Informed consent was obtained for all 22q11.2 participants, as well as for all participants in the comparison cohorts (Institutional Review Board protocols #13-101307, #09-007275, #07-005689, and #10-007622).

### Procedures

We collected data from record review, questionnaires administered remotely, and, for a subset, an autism-specific evaluation. Record review included the participant’s electronic health record at CHOP whenever possible, as well as external medical and educational records (e.g., IEP evaluations) provided by families for individuals who did not receive routine medical care at our institution.

#### Medical record and developmental history review

Medical and developmental history was obtained from a questionnaire completed by the participant. A licensed pediatrician and medical geneticist (TLW) reviewed clinic notes, progress reports, radiology reports, laboratory reports, etc. in each participant’s record to confirm key components reported by participants. Psychiatric and neurodevelopmental diagnostic history was documented in this process as it is routinely collected during clinical visits. Families were contacted by phone to resolve questions or discrepancies.

#### ASD diagnostic classification

##### Sources of diagnostic information

Given that our hypotheses concerned rates of ASD, particular care was given to the ASD classification process. We assigned diagnostic status after a thorough record review of clinical, research, and educational records provided by families and available in the CHOP electronic health record. Participants differed in the frequency with which they received documented CHOP care. Continuous longitudinal data from CHOP developmental pediatricians and psychiatrists existed for individuals who lived locally, whereas records of individuals who lived further away or moved sometimes contained only the initial “22q and You” clinic evaluation. Participants were also asked to provide external medical and educational records.

All families were invited for an in-person ASD evaluation using the Autism Diagnostic Observation Schedule (ADOS and ADOS-2), parent interview, and IQ testing to complete a DSM-5 (*Diagnostic and Statistical Manual of Mental Illness*, 5th edition) checklist [35, 36]. However, since many of our families lived far away, this proved infeasible for a large percentage of the cases. Families who could not complete an in-person evaluation were invited for an hour-long parent phone interview with a clinician asking follow-up questions to Social Communication Questionnaire, Lifetime (SCQ) responses to complete an accurate DSM-5 checklist [35, 36].

##### “ASD” group

We assigned participants to the “ASD” group if there was documentation of an ASD diagnosis (*n* = 5 deletions, *n* = 1 duplication). Five individuals had a diagnostic evaluation in their record; one did not, but had frequent references to the ASD diagnosis throughout the record. All participants scored above threshold (15) on the SCQ.

##### “No ASD” group

We assigned “No ASD” (*n* = 20 deletions, *n* = 8 duplications) if ASD had been considered but specifically ruled out (*n* = 13 deletions, *n* = 3 duplications), or if there was no indication of ASD concerns in the available records (*n* = 7 deletions, *n* = 5 duplications). Two individuals (both LCR-B to D deletions) were excluded because a referral for an ASD evaluation had been recommended recently but not completed.

The *absence* of parental or professional concern about ASD is not routinely documented. Thus, we further investigated this group to determine whether there was a true absence of concern, or a lack of information. We studied parent/spouse report, provider report, behavioral descriptions, and referral history. The 22q clinic routinely refers to developmental behavioral pediatrics or psychiatry if parents indicate relevant concerns during intake, but parents without those concerns would not have had these appointments scheduled. The 22q clinic also routinely questions parents regarding developmental history and previous concern of psychiatric diagnoses from school or medical professionals, as this patient population is at high risk for psychosis and other psychiatric disorders. Any concerns and prior assessments are documented in detail. Therefore, we feel confident that families were routinely asked about developmental concerns and thus that a lack of referrals and text about concerns is a reasonably robust indicator of a lack of ASD concerns.

#### Neuropsychiatric questionnaires

We collected neuropsychiatric questionnaire data from participants under age 18. Questionnaires included a measure of adaptive functioning (Vineland Adaptive Behavior Scales–2nd Edition, “Vineland-II,” completed for participants 0–18 years old [37]), a screener for psychiatric disorders based on DSM-IV checklists (Child and Adolescent Symptom Inventory-4R, “CASI-4R,” completed for participants 5–18 years old depending on disorder [38]), and two measures of social behavior and autistic symptoms (Social Communication Questionnaire––Lifetime, “SCQ,” completed for participants four and above [39], and the Social Responsiveness Scale or Social Responsiveness Scale, 2nd edition, “SRS-2” for participants 2.5–18 years old [40, 41]). Every questionnaire offers excellent psychometric properties, and all but the Social Communication Questionnaire provide standardized scores based on a large, representative norming sample. Please see Additional file 1 for characteristics of subsamples that completed each questionnaire.

#### Analysis

##### ASD rate

To test our hypothesis that the LCR-A to LCR-B region might confer increased risk of ASD in 22q11.2 duplication and deletion syndromes, we compared ASD rates among individuals whose deletion affected the LCR-A to LCR-B region (“AB/AC group”: LCR-A to B, or LCR-A to C) to individuals whose deletion did not affect the LCR-A to LCR-B region (“BD/CD group”: LCR-B to D, and LCR-C to D). Thus, our first analysis compared the AB/AC group to the BD/CD group for deletions only. In a second, more conservative analysis, we compared only individuals with deletions of LCR-A to B to those with LCR-B to D (excluding cases with deleted LCR-A to C or LCR-C to D) to match the groups on approximate size and number of genes in the deletion.

Rates were compared using a one-tailed Fisher’s exact test to account for cells with *n* < 5. An odds ratio (OR) cannot be computed when certain cells contain 0 observations; in these cases, we present 95% confidence intervals and *p* values from Fisher’s exact test and effect sizes as chi-square statistics.

Our sample included no individuals with nested duplications involving LCR-A to B (i.e., no “AB/AC” group for duplications). Thus, we compared the BD/CD duplication group to individuals with the classic LCR-A to D duplication, which does involve LCR-A to B. These results are provided for descriptive purposes only due to the sample size of the nested duplications, which although is one of the largest reported, remains quite small .

##### Psychiatric symptoms (standardized questionnaires)

In our dimensional analysis of psychiatric symptoms using questionnaire data, we analyzed raw scores on the SCQ, age-normed scores on the Vineland-II and SRS-2, and symptom composite scores on the CASI-4R. For deletions, we compared individuals in the “BD/CD” group to the AB/AC group . For duplications, we compared individual in the BD/CD group to the comparison cohort of classic duplications because our sample included no AB/AC duplications. We also compare the AB/AC deletion group to the classic deletion group as this information might prove directly useful clinically. Our interpretations focus on the size of the effect and its confidence interval, as opposed to inferential statistics, to avoid making overly strong statements based on a small sample, as suggested by many recent position papers, e.g.*,* Button et al. [42] and Cumming et al. [43]. We present the effect sizes for each analysis and make our data available upon request so that the data generated here can be leveraged in any future meta-analyses to test our hypothesis directly.

##### Medical and psychiatric diagnoses

We present rates of psychiatric and medical comorbidities by nested region separately for individuals who did and did not receive recommended screening. All analyses are descriptive and for characterization purposes only. Statistical significance was not tested due to small sample sizes within each nested region.

## Results

### Higher rates of ASD when LCR-A to B involved

We observed a trend toward a higher rate of ASD among probands with deletions in the AB/AC group (41.7%, or 5 in 12 individuals with LCR-A to B, or LCR-A to C) compared to the BD/CD group (0%, or 0 in 8 individuals with LCR-B to D, or LCR-C to D; χ = 4.4, *p* = 0.051, CI 0.99, Inf; see Table 3). In a more conservative analysis that matched groups on approximate size of deleted region, we continued to observe similar rates of ASD within each group (44.4%, or 4 of 9 individuals with deletions of LCR-A to B, and 0%, or 0 in 6 individuals with deletions of LCR-B to D; χ = 3.64, *p* = 0.092, CI 0.702, Inf). The rate of ASD did not change meaningfully when related individuals were included to increase sample size; the increased sample size provided more statistical power and revealed significant results (*n* = 25; 38.5% rate in AB/AC group, 0% in BD/CD group; χ = 5.77, *p* = 0.024, CI 1.39, Inf). Thus, the LCR-A to B region may confer increased risk of ASD diagnosis, but a larger sample without related individuals is needed to confim.

Among duplications, individuals with the classic and BD/CD duplications showed similar rates of ASD (24.1% rate or 7 of 29 in classic group, 20% rate or 1 of 5 in BD/CD; OR = 0.79, *p* = 0.764, CI 0.03, Inf). Results did not change meaningfully when related individuals were included to increase sample size (21.4% rate in classic group, 11.1% rate or 1 of 9 in BD/CD; OR = 0.40, *p* = 0.65, CI 0.02, Inf), but this analysis in particular would benefit from a larger sample.

**Fig. 3:**
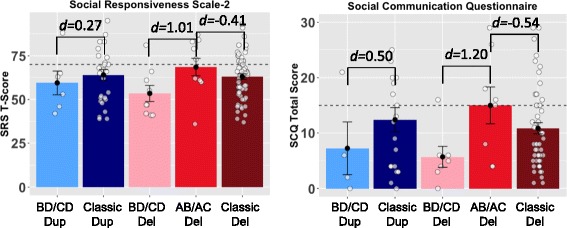
Individuals with deleted LCR-A to B show higher levels of autistic symptoms. This figure depicts group means, standard errors, and the effect size of differences on two quantitative measures of autistic symptoms, the Social Responsiveness Scale-2 and the Social Communication Scale, Lifetime. Each point depicts one individual. Dashed lines indicate the threshold above which an individual is considered to screen positive for autism and warrant further evaluation. The groups with involvement of LCR-A to B show higher levels of social impairment, with large effect sizes for deletions and small to medium effect sizes for duplications. Effect sizes are not significant due to small samples (see Table 4). The AB/AC deletion group includes five individuals diagnosed with autism; the BD/CD deletion group includes zero. Abbreviations: *AB/AC* deletion spanning LCR-A to LCR-B or LCR-A to LCR-C; *BD/CD* deletion or duplication spanning LCR-B to LCR-D or LCR-C to LCR-D; *d* Cohen’s *d* effect size; *del* deletion; *dup* duplication; *LCR* low-copy repeat region; *SCQ* Social Communication Questionnaire, Lifetime; *SRS* Social Responsiveness Scale

Our categorical analysis was supported by quantitative reports of autistic symptoms in the SRS-2 and SCQ (see Fig. 3). A subset of each group (BD/CD deletions, AB/AC deletions, BD/CD duplications, classic duplications, classic deletions) completed the SCQ, including both individuals with and without ASD diagnoses. For deletions, the BD/CD group showed less autistic symptoms than the AB/CD group with large effect sizes (*d*’s of 1.01 and 1.20). For duplications, the difference was small-to-medium (*d*’s of 0.27 and 0.50) between the BD/CD group and the classic group. No effects reached statistical significance (see Table 4)

.

### Moderately lower adaptive and social functioning when AB region involved

**Fig. 4:**
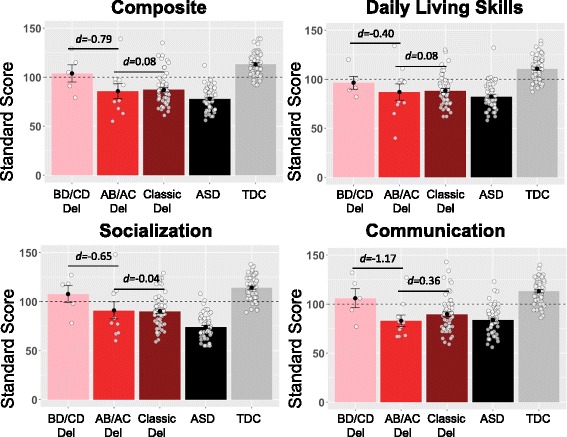
Individuals with deleted LCR-A to B show modestly lower levels of adaptive functioning on the Vineland-II Adaptive Behavior Scales. This figure depicts group means, standard errors, and the effect size of differences on the Vineland-II, a measure of adaptive behavior. Each point depicts one individual). Higher scores on the Vineland-II indicate higher levels of functioning across the three domains and composite score, and standard scores are age-normed such that 100 (represented by the dashed line) indicates average. The AB/AC deletion group shows more impairment than the BD/CD nested deletions that do not involve LCR-A to B, with medium to large effect sizes; the AB/AC group also shows similar levels of impairment to the classic deletion group (deletion spanning LCR-A to D), with very small effect sizes. Abbreviations: *AB/AC* deletion spanning LCR-A to LCR-B or LCR-A to LCR-C, *ASD* individuals with non-syndromic autism spectrum disorder, *BD/CD* deletion spanning LCR-B to LCR-D or LCR-C to LCR-D, *d* Cohen’s d effect size, *del* deletion, *LCR* low copy repeat region, *TDC* typically developing controls

We computed effect sizes for differences in autistic symptoms, psychiatric symptoms, and adaptive behavior skills (see Figs. 3 and 4, Table 4, Additional file 2). For duplications, the differences were usually small between the BD/CD group and the classic duplication group (see Table 4, “Classic Duplication” rows). For deletions, the BD/CD group showed less impairment than the AB/AC group across most measures with medium or large effect sizes that did not reach statistical significance. We also calculated effect sizes for group differences between the AB/AC deletions and classic AD deletion groups and observed small or medium differences (see Table 4, "Classic Deletion" rows). We observed negligible differences between these two groups on most adaptive functioning scales. The classic deletion group showed slightly lower levels of autistic symptoms compared to the AB/AC group––small to medium effect sizes on the SRS-2 and SCQ––that were not statistically significant.

**Table 4:** Group means and effect sizes of group differences on neuropsychiatric questionnaires

	Number	Mean (SD)	*d*	95% confidence interval
SRS-2 T score		50 (10)		
BD/CD deletion	9	53.4 (13.6)		
AB/AC deletion	10	68.5 (15.7)	1.01	(− 0.07, 2.11)
Classic deletion	61	63.0 (12.6)	− 0.41	(− 1.11, 0.27)
BD/CD duplication	6	59.5 (16.6)		
Classic duplication	28	64.0 (16.3)	0.27	(− 0.67, 1.22)
SCQ raw total		Cutoff 15		
BD/CD deletion	7	5.7 (4.9)		
AB/AC deletion	8	15.0 (9.3)	1.20	(− 0.11, 2.52)
Classic deletion	52	10.8 (7.3)	− 0.54	(− 1.32, 0.23)
BD/CD duplication	4	7.2 (9.5)		
Classic duplication	22	12.4 (10.3)	0.50	(− 0.67, 1.68)
Vineland composite		100(15)		
BD/CD deletion	5	103.8 (19.6)		
AB/AC deletion	10	85.7 (24.0)	− 0.79	(− 2.11, 0.52)
Classic deletion	57	87.1 (15.9)	0.08	(− 0.61, 0.78)
BD/CD duplication	6	92.6 (18.7)		
Classic duplication	27	89.4 (19.4)	− 0.16	(− 1.11, 0.78)
Vineland communication		100(15)		
BD/CD deletion	5	106.0 (21.7)		
AB/AC deletion	10	83.0 (18.5)	− 1.17	(− 2.55, 0.20)
Classic deletion	58	89.7 (18.1)	0.36	(− 0.32, 1.06)
BD/CD duplication	6	92.1 (20.2)		
Classic duplication	27	91.2 (18.5)	− 0.04	(− 0.99, 0.90)
Vineland daily living		100(15)		
BD/CD deletion	5	96.4 (14.6)		
AB/AC deletion	10	86.9 (26.2)	− 0.40	(− 1.69, 0.87)
Classic deletion	57	88.4 (15.1)	0.08	(− 0.60, 0.78)
BD/CD duplication	6	91.8 (23.0)		
Classic duplication	28	93.6 (21.2)	0.08	(− 0.86, 1.02)
Vineland socialization		100(15)		
BD/CD deletion	5	107.6 (19.6)		
AB/AC deletion	10	91.0 (27.4)	− 0.65	(− 1.96, 0.65)
Classic deletion	57	90.1 (16.5)	− 0.04	(− 0.74, 0.64)
BD/CD duplication	6	98.1 (18.3)		
Classic duplication	27	90.8 (21.2)	− 0.35	(− 1.30, 0.60)
CASI ADHD				
BD/CD deletion	2	2.5 (1.3)		
AB/AC deletion	7	3.4 (1.4)	0.63	(− 1.56, 2.83)
Classic deletion	43	2.7 (1.2)	− 0.52	(− 1.36, 0.32)
BD/CD duplication	4	2.7 (2.1)		
Classic duplication	20	2.8 (1.3)	0.09	(− 1.09, 1.27)
CASI anxiety				
BD/CD deletion	2	1.3 (0.7)		
AB/AC deletion	7	1.9 (1.1)	0.49	(− 1.68, 2.67)
Classic deletion	43	2.3 (1.3)	0.30	(− 0.53, 1.14)
BD/CD duplication	4	1.9 (1.5)		
Classic duplication	20	1.8 (1.6)	− 0.05	(− 1.24, 1.12)
CASI ASD				
BD/CD deletion	1	n/a		
AB/AC deletion	4	1.0 (1.0)	n/a	n/a
Classic deletion	37	0.5 (0.4)	− 0.83	(− 1.94, 0.27)
BD/CD duplication	4	0.6 (0.4)		
Classic duplication	16	0.8 (0.8)	0.26	(− 0.98, 1.50)
CASI Schizoaffective				
BD/CD deletion	1	n/a		
AB/AC deletion	3	0.5(0.5)	n/a	n/a
Classic deletion	6	0.5(0.2)	− 0.09	(− 0.78, 0.61)
BD/CD duplication	0	n/a		
Classic duplication	4	0.5(0.2)	n/a	n/a
CASI behav. regulation				
BD/CD deletion	2	0.9 (0.4)		
AB/AC deletion	7	0.9 (0.6)	0.06	(− 2.08, 2.21)
Classic deletion	43	1.0 (0.6)	0.16	(− 0.67, 1.00)
BD/CD duplication	4	0.3 (0.3)		
Classic duplication	20	1.0 (0.8)	0.78	(− 0.42, 2.00)
CASI Depression				
BD/CD deletion	2	0.4 (0)		
AB/AC deletion	7	0.6 (0.7)	0.33	(− 1.82, 2.50)
Classic deletion	43	0.5 (0.7)	− 0.15	(− 0.99, 0.67)
BD/CD duplication	4	0.2 (0.4)		
Classic duplication	20	0.4 (0.4)	0.48	(− 0.70, 1.68)

Group means on neuropsychiatric questionnaires. We show 95% confidence intervals of effect sizes as Cohen’s *d*, which can be interpreted as follows: 0.2 as small, 0.5 as medium, and 0.8 as large [54]. Means and standard deviations for each group are presented, as well as the mean and SD for each measure to aid in interpretation. We derived SRS T-scores using the updated SRS-2 norms for all participants, regardless of the version the participant completed. We averaged CASI-4R raw item scores on similar subscales into composites instead of using T-scores because we encountered a strong ceiling effect when using CASI-4R T-scores because CASI-4R norms collapse all high raw scores into a T-score of 70. Thus, population-normed means and standard deviations are not available for comparison for these averaged composites. For example, all items from the dysthymia subscale and major depression subscales were averaged into a “Depression” composite, after accounting for the number of items in each subscale so that both scales were weighted equally in the composite. The composites are interpreted as “3” indicating that on average, the parent endorsed symptoms in the domain as occurring “very often,” 2 as “often,” 1 as “sometimes,” and 0 as “never”

*Vineland* Vineland Adaptive Behavior Scales, 2nd Edition; *CASI* Child and Adolescent Symptom Inventory-4R; *SCQ* Social Communication Questionnaire; *SRS-2* Social Responsiveness Scale, 2nd Edition; *n/a* not applicable

### Increased rates of psychiatric disorders

In individuals with nested duplications or deletions, we observed elevated rates compared to population means in nearly every psychiatric disorder reported, including ADHD, OCD, ODD and related behaviors, depression, language disorders, global developmental delay, and intellectual disability. See Table 5 for observed rates of disorders by type of nested deletion or duplication.

**Table 5:** Psychiatric disorder rates from parent and adult self-report and chart review

	Total (*n*)	No eval	Had eval	GDD	Lang Dx	ADHD	ID	ODD	OCD	Anxiety	MDD
AB/AC deletion	13	7.7%	92.3%	30.8%	0.0%	53.8%	0.0%	7.7%	23.1%	23.1%	0.0%
A-B deletion	10	10%	90%	30%	0%	50%	0%	10%	20%	20%	0%
< 3 yrs	1	1	0	0	0	0	0	0	0	0	0
3–14 yrs	8	0	8	2	0	4	0	0	1	1	0
15+ yrs	1	0	1	1	0	1	0	1	1	1	0
A-C deletion	3	0%	100%	33.3%	0%	66.7%	0%	0%	33.3%	33.3%	0%
3–14 yrs	1	0	1	1	0	0	0	0	0	0	0
15+ yrs	2	0	2	0	0	2	0	0	1	1	0
BD/CD deletion	12	33.3%	66.7%	16.7%	8.3%	8.3%	25%	8.3%	25%	25%	0%
B-D deletion	8	12.5%	87.5%	37.5%	0%	12.5%	12.5%	0%	0%	25%	25%
< 3 yrs	1	1	0	0	0	0	0	0	0	0	0
3–14 yrs	5	0	5	3	0	1	1	0	0	0	0
15+ yrs	2	0	2	0	0	0	0	0	0	2	2
C-D deletion	4	75%	25%	0%	0%	25%	0%	25%	25%	25%	25%
< 3 yrs	2	2	0	0	0	0	0	0	0	0	0
15+ yrs	2	1	1	0	0	1	0	1	1	1	1
BD/CD duplication	9	33.3%	66.6%	22.2%	0%	0%	44.4%	0%	11.1%	11.1%	11.1%
B-D duplication	7	42.9%	57.1%	28.6%	14.3%	28.6%	0%	0%	0%	14.3%	14.3%
< 3 yrs	1	1	0	0	0	0	0	0	0	0	0
3–14 yrs	3	0	3	2	1	1	0	0	0	1	0
15+ yrs	3	2	1	0	0	1	0	0	0	0	1
C-D duplication	2	0%	100%	100%	0%	0%	0%	0%	0%	0%	0%
3–14 yrs	2	0	2	2	0	0	0	0	0	0	0
Total sample	34	23.5%	76.5%	32.3%	2.9%	32.3%	2.9%	5.9%	11.8%	20.6%	11.8%

We observed elevated rates of psychiatric diagnoses among individuals with nested duplications or deletions relative to population base rates using parent- and self-report data confirmed in medical records. Among the full sample, 77% had received a psychiatric evaluation. The most commonly reported diagnoses in our sample included ADHD and Global Developmental Delay (GDD), which may reflect the sample’s skew toward younger ages (see Table 2 for sample characteristics). We present rates for group totals, and we present *n*’s for age bins based roughly on when documentation of diagnosis would be expected (i.e., GDD and language disorders are frequently diagnosed before age 3, ADHD and ID are usually diagnosed in childhood after age 3, and depression and anxiety frequently onset during adolescence or adulthood) to facilitate interpretation of overall group rates because rates for disorders that frequently appear in adolescence (e.g., anxiety and depression) are likely underestimates

Abbreviations: *Eval* evaluation; *ADHD* attention deficit/hyperactivity disorder; *ID* intellectual disability; *OCD* obsessive compulsive disorder; *GDD* Global Developmental Delay; *Lang Dx* language disorder, receptive or expressive; *ODD* oppositional defiant disorder; *MDD* major depressive disorder; *Del* deletion; *Dup* duplication; *yrs* years

### Higher rates of medical comorbidities

We documented presence or absence of having received an appropriate screening test, and whether or not an abnormality was identified, in individuals with nested deletions and duplications between LCR-A and D (see Table 6). In order to calculate conservative estimates for the prevalence of each medical comorbidity in each group, we report both the percentage of screened individuals and the percentage of total individuals.

**Table 6:** Medial comorbidities in individuals with nested deletion or duplication of 22q11.2

	A to B deletion (*n* = 11)	A to C deletion (*n* = 4)	B to D deletion (*n* = 14)	B to D duplication (*n* = 8)	C to D deletion (*n* = 4)	C to D duplication (*n* = 2)
Audiologic						
Audiogram	11	4	14	8	4	2
Abnormal	6	1	2	2	0	1
Abnormal Rate^a^	55%	25%	14%	25%	0%	50%
Abnormalities	CHL	CHL	SNHL	CHL, CSNHL	n/a	CHL
Cardiac						
Echocardiogram	9	4	11	5	2	1
Abnormal	7	3	7	1	1	0
Abnormal Rate^a^	64% (78%)	75%	50% (64%)	12% (20%)	25% (50%)	0%
Abnormalities	Enlarged PA, VR, PS, TOF with PS, IAA with ARSCA, TR, PDA	TA, RAA with ALSCA, dilated aortic root, VR, ASD/VSD	Aortic root dilation, aneurism of TV, ASD, PDA, PFO, TA, VSD	PFO	TOF with pulmonary valve stenosis	n/a
Endocrine						
Bloodwork	11	4	14	4	4	1
Abnormal	6	2	6	2	0	0
Abnormal Rate^a^	55%	50%	43%	25% (50%)	0%	0%
Abnormalities	Hypocalcemia hypothyroidism low vitamin D (each category *n* = 2)	Hypocalcemia	Hypocalcemia (*n* = 2), diabetes mellitus, borderline HbA1C, low growth factors, low vitamin D, hypothyroidism	Borderline abnormal thyroid function tests, neonatal hypoglycemia	n/a	n/a
GI						
Symptom screen	11	3	13	6	3	2
Abnormal	7	2	12	5	1	2
Abnormal rate^a^	64%	50% (67%)	86% (92%)	63% (83%)	25% (33%)	100%
Abnormalities	GERD (*n* = 7), constipation (*n* = 4), anal atresia (*n* = 1), feeding tube (*n* = 2)	Constipation (*n* = 2), GERD	GERD (*n* = 11), constipation (*n* = 9), feeding tube (*n* = 4)	GERD (*n* = 4), eosinophilic esophagitis, feeding tube	GERD, chronic constipation	GERD (*n* = 2), constipation, feeding tube
Hematologic						
CBC completed	11	4	14	8	4	2
Cytopenias	3	3	2	0	0	0
Abnormal Rate^a^	27%	75%	14%	0%	0%	0%
Immune						
Bloodwork	9	4	11	4	4	2
Abnormal	2	2	4	1	2	0
Abnormal Rate^a^	18% (22%)	50%	29% (36%)	13% (26%)	50%	0%
Abnormalities	Low Ig	Low Ig, T cell lymphopenia, inadequate vaccine titers	Low Ig (*n* = 2), absent thymus, inadequate vaccine response	Low Ig	Low Ig, recurrent MRSA infections, inadequate vaccine response	n/a
Neurologic						
Seizures	3	0	2	0	0	0
% reported	27%	0%	14%	0%	0%	0%
MRI	8	2	10	4	0	0
Abnormal MRI	3	1	3	2	n/a	n/a
Abnormal Rate^a^	27% (38%)	25% (50%)	21% (30%)	25% (50%)	0%	0%
MRI findings	Chiari 1, white matter lesions, pachygyria	Minimal bilateral congenital optic nerve hypoplasia	Chiari 1, hypoplastic corpus callosum, polymicrogyria	Prominent ventricles, subarachnoid spaces, choroid plexus cysts	n/a	n/a
Ophthalmologic						
Ophtho exam	9	4	11	3	4	1
Abnormal	3	2	5	1	0	0
Abnormal rate^a^	27% (33%)	50%	45%	13% (33%)	n/a	n/a
Abnormalities	Astigmatism, exophoria, nystagmus	Strabismus, minimal ONH	Anisocoria, iris coloboma, ONH nystagmus (*n* = 2), retinal detachment, strabismus (*n* = 3)	Amblyopia	n/a	n/a
Palate						
Clinical eval.	9	4	12	6	2	2
Abnormal	8	3	4	1	0	1
Abnormal rate^a^	89% (73%)	75% (27%)	33% (29%)	17% (13%)	0%	50%
Abnormalities	SMCP (*n* = 3) VPI (*n* = 8)	SMCP (n = 1), VPI (*n* = 3)	SMCP (*n* = 2), VPI (*n* = 4)	High arched palate with small uvula	n/a	VPI
Renal						
Ultrasound	9	2	9	6	2	1
Abnormal	3	0	1	4	1	0
Abnormal rate^a^	27% (33%)	0%	7% (11%)	50% (67%)	25% (50%)	0%
Abnormalities	Bilateral pelviectasis, nephrocalcinosis hydronephrosis	n/a	Medullary nephrocalcinosis	Duplicated collecting system, small kidneys (*n* = 3)	Solitary, low-lying kidney	n/a
Spine						
Screening x-rays	6	4	4	2	1	1
Abnormal	6	3	2	1	1	0
Abnormal Rate^a^	55% (100%)	38% (75%)	14% (50%)	13% (50%)	25% (100%)	0%
Abnormalities	Hypoplastic vertebra (*n* = 2), vertebral fusion (*n* = 4), extra lumbar vertebra	Fusion of C2-C3, kyphoscoliosis, thickened spinous process of C2	Scoliosis, C2-C3 fusion and dysmorphic dens, upswept C2	Hemivertebra at T9, absent rib	6 thoracic ribs and 6 lumbar vertebrae	n/a

The total number of patients in each group is designated in column headings. Each screened organ system is listed along with the number of patients who received the screening recommended for patients with classic 22q11.2 deletions and duplications. ^a^We present the rate of patients with abnormal findings as a percentage of total patients. Many patients did not receive all recommended screening; when not all patients were screened, we use parentheses to note the percentage of patients with abnormal findings ofthose who received screening

Abbreviations: *ARSCA* aberrant right subclavian artery, *ALSCA* aberrant left subclavian artery, *ASD* atrial septal defect (in cardiac row only, in remainder of manuscript ASD refers to autism spectrum disorder), *CHL* conductive hearing loss, *C/SNHL* mixed conductive and sensorineural hearing loss, *GERD* gastroesophageal reflux disease, *HbA1C* hemoglobin A1C, *IAA* interrupted aortic arch, *Ig* immunoglobulins, *MRI* magnetic resonance imaging, *MRSA* methicillin-resistant *Staphylococcus aureus*, *ONH* optic nerve hypoplasia, *PA* pulmonary artery, *PDA* patent ductus arteriosus, *PFO* patent foramen ovale, *PS* pulmonic stenosis, *SMCP* submucous cleft palate, *SNHL* sensorineural hearing loss, *TA* truncus arteriosus, *TR* tricuspid regurgitation, *TOF* Tetralogy of Fallot, *TV* tricuspid valve, *VPI* velopharyngeal insufficiency, *VR* vascular ring, *VSD* ventricular septal defect, *n/a* not applicable

### Case study 1

#### Isolating specific genes: an individual with ASD and tiny duplication involving *RANBP1* and *COMT*, not *TBX1*

One individual in our sample came to attention of clinical geneticists due to autism spectrum disorder and was found to have a small, 300 kb microduplication within the LCR-A to B region that included *RANBP1* and *COMT* but not *TBX1*. Detailed clinical evaluation and all recommended medical screening for individuals with 22q11.2-related disorders revealed none of the medical issues or dysmorphic features characteristic of the syndrome. However, the individual met diagnostic criteria for ASD, anxiety, and ADHD after evaluation by a neurodevelopmental pediatrician and standardized neuropsychiatric evaluation. The inheritance of this microduplication is unknown because parental testing was not possible. To our knowledge, no relatives carry an autism diagnosis but none have received formal evaluation. The individual’s SNP array showed no other pathogenic variants. This individual was not included in group analyses because the duplication did not encompass the full LCR-A to B region.

### Case study 2

#### The role of background genetics: a family with LCR-B to D duplication and distal E-F duplication and autism and face processing deficits

The only individual in our analyses with autism in the BD/CD group carried a duplication of LCR-B to D. She had one sibling with the same LCR-B to D duplication and two siblings with a duplication of *TOP3B* (in a small region between LCR-E and F). One of the siblings with the *TOP3B* duplication had a history of an autism diagnosis but did not currently present with significant autism symptoms. Furthermore, the proband and the sibling with LCR-B to D duplication both showed decreased face processing abilities on the Benton Facial Recognition Test (mildly impaired in the proband, clinically impaired in the sibling). Face processing difficulties have not been reported in 22q syndromes before, and we do not posit that they are central to the syndromes, but rather that the family history of possible ASD and the genetic complexity of the family raises the question that other genetic factors may have contributed to the proband’s autism. Future studies of autism in nested 22q11.2 should evaluate family members for ASD and prosopagnosia, and evaluate probands for phenotypes seen in other family members, to better understand the contribution of background genetics.

## Discussion

To our knowledge, this study includes the largest group of individuals with nested deletions and duplications of 22q11.2 to be compared prospectively to classic deletions and duplications with standardized measures. These data suggest that individuals with deletion of the LCR-A to B region may have a higher rate of ASD (39–44%) than those without involvement (0%); the pattern was not replicated for duplications. Taken in conjunction with case study 1, these findings are consistent with our hypothesis that LCR-A to B may confer risk for ASD in 22q11.2 related disorders. However, we offer this evidence as preliminary support that requires further exploration with additional samples.

It is notable that the nested deletions of all individuals with ASD involved LCR-A to B and that we observed negligible differences between this group and the classic deletion spanning LCR-A to D in adaptive functioning. These results suggest that LCR-A to B could be contributing to the autistic phenotype in individuals with classic 22q11.2DS, as well as to decreased adaptive functioning. It is also notable that we observed no duplications of LCR-A to B or LCR-A to C in our full sample of 43 individuals, although such individuals are mentioned in much larger studies [44]. Thus, it remains to be tested in larger samples whether these individuals are as likely to present with ASD as those with the classic A-D duplication.

### Implications for medical screening

Prior studies have suggested that individuals with nested deletions have similar types of medical problems to those with classic deletions and should receive similar clinical treatment. The medical chart review of our patients supported this hypothesis. It also suggested that our patients are representative of other previously reported patients with nested deletions with regard to the frequency and types of medical problems. It is notable that there appeared to be fewer medical problems in individuals with LCR-C to D. However, this region is much smaller, encompassing fewer genes than the other regions. In size and total number of genes, LCR-A to LCR-B and LCR-B to LCR-D are roughly equivalent, and the rates of medical comorbidities are similar. We also observed higher rates of some medical comorbidities in several of the nested groups as compared to individuals with full LCR-A to LCR-D deletions (e.g., cervical spine anomalies in 100% of screened individuals with LCR-A to LCR-B deletion), but our sample sizes are too small to determine if this is due to chance or truly represents a higher risk subgroup. We were somewhat surprised to find that many patients had not completed portions of the recommended medical screening for individuals with 22q11.2-related disorders. It is unclear if this is due to a perception by providers that individuals with nested deletions do not need as aggressive screening as those with full deletions or duplications. Overall, we observed rates of each of the medical comorbidities in the LCR-A to LCR-B and LCR-B to LCR-D subgroups that are comparable to rates in individuals with full LCR-A to LCR-D deletions or duplications. Although the rate of medical problems appears lower in the LCR-C to LCR-D deletion and duplication groups, the sample sizes are extremely small, and therefore, no strong conclusions can be made about the validity of an altered screening protocol for these patients.

### *RANBP1* as a potential ASD candidate gene

The LCR-A to B region associated with ASD risk in our sample involves approximately 25 genes, including *COMT*, *PRODH*, and *TBX1*. Prior research implicates the interaction of low activity *COMT* and *PRODH* alleles in ASD risk [28, 29]. Other genes in the region may also confer ASD risk, and indeed, the risk could be additive. We propose another possible candidate gene, Ran-binding protein 1 (*RANBP1)*, which could not be examined given our study design with multiple candidate genes in the LCR-A to B region, warranting further investigations. We base this speculation on five circumstantial pieces of evidence.

First, we cite the involvement of *RANBP1* in the metabotropic glutamate receptor (mGluR) gene network [44], which is disrupted in two other syndromic forms of ASD, fragile X syndrome and tuberous sclerosis complex [45]. Second, we previously observed a 10-fold increase in ASD rate among individuals with 22q11.2DS with a “second hit” in an mGluR network gene compared to individuals without a “second hit” (5 affected in 25 individuals with 22q11.2 compared to 1 in 50) [30]. Third, two teratogens associated with increased rates of ASD––valproate and thalidomide––both decrease expression of *RANBP1* [46, 47, 48]. Fourth, the important link between *RANBP1* and expression in human brains was demonstrated by Meechan et al. [49], who showed higher *RANBP1* expression in developing fetal brains compared to adult brains during a peak in neurogenesis. Finally, several studies in the 22q11.2 animal literature highlight *RANBP1* as important for neural development in 22q11.2 (e.g., [49–51]). Taken together, these disparate pieces of literature converge on a role of *RANBP1* in brain development, and potentially in ASD. Like other genes and gene families recently associated with ASD, *RANBP1* serves a general function within the cell (metabolizing GTP and regulating material transport to the nucleus [52]). *RANBP1* has not been identified previously as an ASD candidate gene in large ASD studies; of the approximately 25 genes in the 22q11.2 LCR-A to LCR-B region, previous genome-wide association studies or whole exome sequencing studies have identified *PRODH* as a candidate gene with suggestive evidence and *TBX1* and *GNB1L* as candidate genes with minimal evidence at this time (SFARI gene database https://gene.sfari.org/database/human-gene/). It is not yet clear whether genes in this region modify ASD risk in the general population or in the context of 22q11.2 syndromes alone.

### Insights from two case studies involving *TBX1* and *RANBP1*

Individuals with very small nested duplications and deletions offer a unique method of studying the associations between isolated regions or genes and individual features of the 22q11.2DS phenotype. In the present study, we could not tease apart the contributions of individual genes to portions of the phenotype, as the LCR-A to B region includes approximately 25 genes. Here, we contrast two case studies, case study 1 and a prior case study by Weisfeld-Adams and colleages [53], each with a very small duplication including either *TBX1* or *RANBP1*, but not both, to provide some insight into the possible relative contributions of *TBX1* and *RANBP1* to the phenotype in a descriptive fashion. Weisfeld-Adams et al. described a patient and sibling with duplication of six genes including *TBX1* but not *RANBP1*. This proband showed complex medical problems, but neither the 19-month-old proband nor the 3-year-old sibling showed any symptoms of autism or neurodevelopmental delay besides mild motor delay. (Although no concern for ASD was noted at 19 months of age, we caution against over-interpretation because ASD can be missed in toddlers when symptoms are not severe. However, by 19 months of age most children with 22q11.2DS show significant delays, little speech, and aloof social behavior, so the lack of delay suggests social development was on course.) In contrast, in case study 1, we described an individual with microduplication involving *RANBP1* but not *TBX1* who had ASD but no medical comorbidities. Both our patient, who had a purely psychiatric phenotype and duplication that *does* involve *RANBP1*, and the case presented by Weisfeld-Adams et al.––a purely medical phenotype that does *not* involve *RANBP1*––provide preliminary suggestive evidence that *RANBP1*, not *TBX1*, specifically might confer risk for ASD and other psychiatric diagnoses. Both microduplications include *COMT* and exclude *PRODH*, so we cannot speculate about the roles of these genes based on case studies.

### Limitations

The two primary limitations of our study lie in the phenotyping and the sample size. This single-site study relied primarily on questionnaires and chart review, supplemented by in-person evaluation when feasible for the family. Thus, the phenotyping, while accurate, could be improved with systematic prospective evaluations. Our sample size was small, owing to the rarity of individuals with nested duplication or deletions in the 22q11.2 region. Our study would benefit from replication with a multi-site study that combines clinics around the world to improve statistical power.

Another limitation includes the unknown role of background genetics. We were unable to account for other contributors to ASD risk, such as common variants or known pathogenic variants occurring outside 22q11.2 that would be identified with whole-exome sequencing, not clinical genetic testing with MLPA and SNP arrays. However, this risk is likely to affect all groups equally. Furthermore, we believe this unknown potential risk is likely to be small compared to the known, larger ASD risk of carrying 22q11.2DS or DupS.

Future directions might involve whole-exome sequencing of 22q11.2 samples to identify other factors that contribute to ASD risk. Such a study should include an analysis leveraging the sequencing of *PRODH*, *COMT*, *RANBP1*, and *TBX1* in individuals with nested 22q11.2 deletions and duplications to isolate the influence of these mutations on the ASD phenotype.

## Conclusions

We present data on medical and psychiatric issues in 44 individuals with nested duplications and deletions within the LCR-A to D region, along with two additional siblings with tiny duplication of *TOP3B*, the largest cohort of this type to be studied prospectively. We found increased rate of ASD among individuals with deleted LCR-A to B, compared to individuals whose nested deletions did not involve that region. We tentatively speculate that *RANBP1* could provide a potential mechanistic explanation for increased rates of ASD based on this finding, our reported case study, environmental ASD risk factors that also alter *RANBP1* expression, *RANBP1*’s role in the mGluR network, and the role of the mGluR network in other syndromic forms of ASD. We also conclude from our observation of the full spectrum of medical issues in each group that at this time, there is insufficient evidence to limit medical screening in individuals with nested duplications or deletions within the 22q11.2 region.

## Additional files

Additional file 1: This file depicts participant characteristics for each questionnaire. (DOCX 26 kb)

Additional file 2: This file depicts higher parent-reported psychiatric symptoms among individuals with nested or classic 22q11.2 duplication or deletions compared to typically developing controls. (DOCX 266 kb)

## Abbreviations

22q11.2DS: 22q11.2 deletion syndrome
22q11.2DupS: 22q11.2 duplication syndrome
ADHD: Attention-deficit/hyperactivity disorder
ADI-R: Autism Diagnostic Interview-Revised
ADOS: Autism Diagnostic Observation Schedule
ASD: Autism spectrum disorder
CASI-4R: Child and Adolescent Symptom Inventory-4R
CHOP: Children’s Hospital of Philadelphia
CNV: Copy number variation
*COMT*: Catechol-O-methyltransferase
DSM: *Diagnostic and Statistical Manual of Mental Disorders*
FXS: Fragile X syndrome
LCR: Low-copy repeat
mGluR: Metabotropic glutamate receptor
MLPA: Multiplex ligation probe amplification
Ran: Ras-related nuclear protein
*RANBP1*: Ran-binding protein 1
SCQ: Social Communication Questionnaire
SNP: Single nucleotide polymorphism
SRS: Social Responsiveness Scale
TDC: Typically developing children
Vineland-II: Vineland Adaptive Behavior Scales-II
