# Supplementary material for: Critical region within 22q11.2 linked to higher rate of autism spectrum disorder
Source: Mol Autism. 2017 Oct 27;8:58. doi: 10.1186/s13229-017-0171-7 (PMC5658953; doi:10.1186/s13229-017-0171-7)
Supplement: Supplementary file 1 — This file depicts participant characteristics for each questionnaire. (DOCX 26 kb) [file 13229_2017_171_MOESM1_ESM.docx]

Additional File 1

Table S1. Descriptive characteristics of participants included in neuropsychiatric questionnaires: Social Communication Questionnaire, Lifetime

| Region | N | Age mean(sd) | Age range | % Male |
| --- | --- | --- | --- | --- |
| **AB/AC del group** | 13 | 14.5 (10.4) | 6-38 | 46% |
| A-B Deletion | 10 | 15.6 (12.9) | 6-38 | 50% |
| A-C Deletion | 3 | 12.7 (6.3) | 6-18 | 33% |
| **BD/CD del group** | 12 | 9.5 (5.2) | 4-18 | 50% |
| B-D Deletion | 8 | 10 (3.5) | 6-14 | 38% |
| C-D Deletion | 4 | 8.9 (7.8) | 4-18 | 75% |
| **Classic AD del** | 70 | 8.7 (3.4) | 4-17 | 77% |
| **BD/CD dup group** | 9 | 7.8 (2.8) | 5-11 | 56% |
| B-D Duplication | 7 | 7.7 (2.2) | 6-9 | 57% |
| C-D Duplication | 2 | 7.9 (4.3) | 5-11 | 50% |
| **Classic AD dup** | 29 | 8.4 (3.2) | 4-14 | 76% |
| **ASD** | 70 | 7.8 (3.3) | 3-14 | 80% |
| **TDC** | 73 | 7.8 (3.5) | 2-14 | 77% |

Table S2. Descriptive characteristics of participants included in neuropsychiatric questionnaires: Social Responsiveness Scale-2

| Region | N | Age mean(sd) | Age range | % Male |
| --- | --- | --- | --- | --- |
| **AB/AC del group** | 13 | 12.4 (10.3) | 2-38 | 46% |
| A-B Deletion | 10 | 12.3 (12.1) | 2-38 | 50% |
| A-C Deletion | 3 | 12.7 (6.3) | 6-18 | 33% |
| **BD/CD del group** | 12 | 16.2 (14.8) | 4-42 | 50% |
| B-D Deletion | 8 | 15.8 (14.9) | 6-42 | 38% |
| C-D Deletion | 4 | 16.7 (16.8) | 4-40 | 75% |
| **Classic AD del** | 70 | 7.7 (3.8) | 2-16 | 77% |
| **BD/CD dup group** | 9 | 6.2 (3.4) | 2-11 | 56% |
| B-D Duplication | 7 | 5.3 (3.1) | 2-9 | 57% |
| C-D Duplication | 2 | 7.9 (4.3) | 5-11 | 50% |
| **Classic AD dup** | 29 | 7.4 (3.5) | 3-14 | 76% |
| **ASD** | 70 | 7.8 (3.3) | 3-14 | 80% |
| **TDC** | 73 | 7.8 (3.5) | 2-14 | 77% |

Table S3. Descriptive characteristics of participants included in neuropsychiatric questionnaires: Vineland Adaptive Behavior Scales-II

| Region | N | Age mean(sd) | Age range | % Male |
| --- | --- | --- | --- | --- |
| **AB/AC del group** | 13 | 11.6 (10.4) | 2-38 | 46% |
| A-B Deletion | 10 | 11.5 (12.3) | 2-38 | 50% |
| A-C Deletion | 3 | 11.7 (5.3) | 6-15 | 33% |
| **BD/CD del group** | 12 | 3 (2.9) | 0-8 | 50% |
| B-D Deletion | 8 | 4.1 (3.3) | 2-8 | 38% |
| C-D Deletion | 4 | 1.3 (1.3) | 0-2 | 75% |
| **Classic AD del** | 70 | 7.3 (4) | 2-16 | 77% |
| **BD/CD dup group** | 9 | 5.7 (2.7) | 2-9 | 56% |
| B-D Duplication | 7 | 5.3 (3.1) | 2-9 | 57% |
| C-D Duplication | 2 | 6.4 (2.2) | 5-8 | 50% |
| **Classic AD dup** | 29 | 7.1 (3.4) | 3-14 | 76% |
| **ASD** | 70 | 7.8 (3.3) | 3-14 | 80% |
| **TDC** | 73 | 7.8 (3.5) | 2-14 | 77% |

Table S4. Descriptive characteristics of participants included in neuropsychiatric questionnaires: Child and Adolescent Symptom Inventory, 4^th^ Edition, Revised

| Region | N | Age mean(sd) | Age range | % Male |
| --- | --- | --- | --- | --- |
| **AB/AC del group** | 13 | 11.6 (10.4) | 2-38 | 46% |
| A-B Deletion | 10 | 11.5 (12.3) | 2-38 | 50% |
| A-C Deletion | 3 | 11.7 (5.3) | 6-15 | 33% |
| **BD/CD del group** | 12 | 3 (2.9) | 0-8 | 50% |
| B-D Deletion | 8 | 4.1 (3.3) | 2-8 | 38% |
| C-D Deletion | 4 | 1.3 (1.3) | 0-2 | 75% |
| **Classic AD del** | 70 | 7.3 (4) | 2-16 | 77% |
| **BD/CD dup group** | 9 | 5.7 (2.7) | 2-9 | 56% |
| B-D Duplication | 7 | 5.3 (3.1) | 2-9 | 57% |
| C-D Duplication | 2 | 6.4 (2.2) | 5-8 | 50% |
| **Classic AD dup** | 29 | 7.1 (3.4) | 3-14 | 76% |
| **ASD** | 70 | 7.8 (3.3) | 3-14 | 80% |
| **TDC** | 73 | 7.8 (3.5) | 2-14 | 77% |

**Abbreviations**: ASD: autism spectrum disorder; d; Cohen’s d effect size; del: typical 22q11.2 Deletion Syndrome involving LCR-A to D, dup: typical 22q11.2 Duplication Syndrome involving LCR-A to D, TDC: typically developing controls; SCQ: Social Communication Questionnaire, Lifetime; SRS: Social Responsiveness Scale
