# Supplementary material for: Critical region within 22q11.2 linked to higher rate of autism spectrum disorder
Source: Mol Autism. 2017 Oct 27;8:58. doi: 10.1186/s13229-017-0171-7 (PMC5658953; doi:10.1186/s13229-017-0171-7)
Supplement: Supplementary file 2 — This file depicts higher parent-reported psychiatric symptoms among individuals with nested or classic 22q11.2 duplication or deletions compared to typically developing controls. (DOCX 266 kb) [file 13229_2017_171_MOESM2_ESM.docx]

Additional File 2

**Title**: Patterns in parent-reported psychiatric symptoms across individuals with classic or nested 22q11.2 duplications or deletions compared to typically developing controls


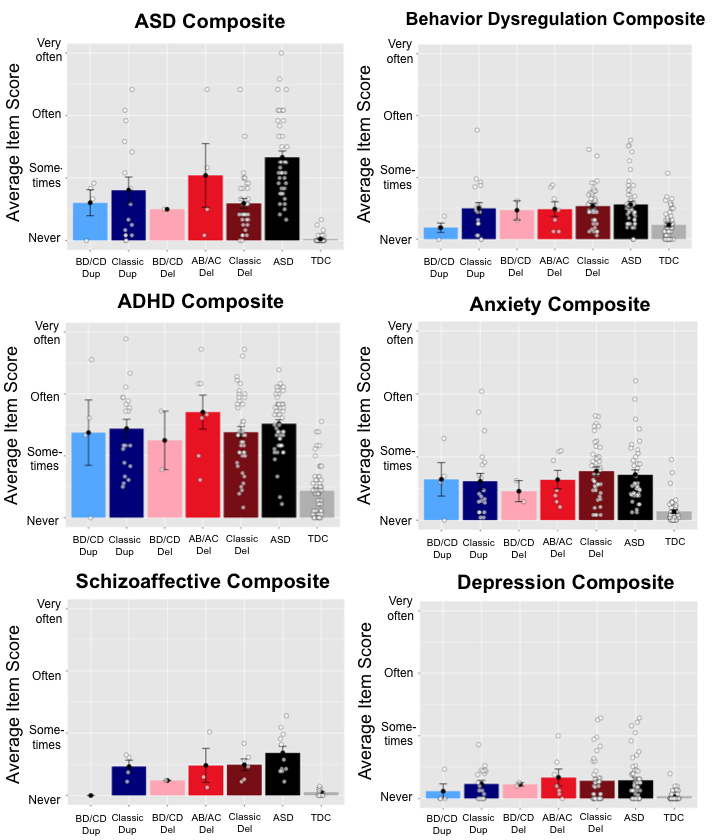


**Caption**: Group means and standard errors on six composite indices of the CASI-4R, a parent-report measure of psychiatric symptoms in DSM-5 diagnoses. Groups include the “BD/CD” duplication (light blue) or deletion (pink) groups (individuals with nested duplication or deletion involving LCR-B to LCR-C or D), the “AB/AC” deletion group in red (individuals with nested deletion of LCR-A to B or C), the “Classic Del” group in dark red (individuals with typical deletion spanning LCR-A to LCR-D), “Classic Dup” group in dark blue (individuals with typical duplication spanning LCR-A to LCR-D), “ASD” group in black (individuals with non-syndromic autism spectrum disorder), and “TDC” group in gray (typically developing children). Higher scores on the CASI-4R indicate higher symptom levels. The “BD/CD” deletion (pink) and duplication (light blue) groups showed similar or lower levels of symptoms compared to the other deletion or duplication groups, respectively (see table 4 for details). All 22q11.2 groups show higher symptom levels than the typically developing controls.

**Abbreviations**: ASD: autism spectrum disorder; CASI-4R: Child and Adolescent Symptom Inventory-4R; del: classic 22q11.2 Deletion Syndrome involving LCR-A to D, dup: classic 22q11.2 Duplication Syndrome involving LCR-A to D, TDC: typically developing controls
